# How parenting styles affect subjective well-being in middle school students? The chain mediating role of family functioning and core self-evaluation

**DOI:** 10.3389/fpsyt.2026.1719614

**Published:** 2026-07-30

**Authors:** Li Fu, Shuxing Shen, Minhui Wen, Liping Zhang, Jiangjie Sun

**Affiliations:** 1 School of Health Care Management, Anhui Medical University, Hefei, China; 2 School of Marxism, Anhui Medical University, Hefei, China

**Keywords:** core self-evaluation, family functioning, middle school students, parenting styles, subjective well-being

## Abstract

**Objective:**

Parenting styles are crucial to adolescent development, yet how they affect subjective well-being (*SWB*) remains unclear. Grounded in ecological systems theory, this study examines the effects of parenting styles on middle school students’ *SWB* and tests the chain mediation of family functioning and core self-evaluation.

**Methods:**

Using cluster sampling, data on parenting styles, family functioning, core self-evaluation, and *SWB* were collected from 542 participants in two middle schools in Anhui Province. Questionnaire data were entered using Epidata 3.1 and exported to SPSS 26.0. Pearson’s correlation analysis was performed using R 4.5.1 to examine the bivariate correlations among all study variables. Mediation analysis was conducted using Mplus 8.3 with maximum likelihood estimation and bootstrap resamples to test the chain mediating effects of family functioning and core self-evaluation between parenting styles and *SWB*.

**Results:**

Positive parenting styles were significantly positively correlated with family functioning, core self-evaluation, and *SWB* (r = 0.45 ~ 0.68, *p* < 0.001), while negative parenting styles showed significant negative correlations with family functioning, core self-evaluation, and *SWB* (r = −0.41~ −0.31, *p* < 0.001). Regression models showed that positive parenting style positively predicted *SWB* (B = 0.411, *p* < 0.05) and negative parenting style negatively predicted *SWB* (B= −0.872, *p* < 0.001). The chain mediation effects of family functioning and core self-evaluation were significant for both positive parenting style (effect value: 0.243; 95% CI [0.135, 0.372]) and negative parenting style (effect value: −0.060; 95% CI [−0.127, −0.020]).

**Conclusion:**

Parenting styles influence middle school students’ *SWB* directly and indirectly through family functioning and core self-evaluation. These findings provide important reference values for enhancing adolescent well-being.

## 1 Introduction

Mental health challenges have escalated into a global public health crisis (1). The adolescent population is disproportionately affected by mental health problems (2), with global evidence indicating widespread mental health disorders among adolescents (3), and rates of suicidal, self-harming, and other behaviors among adolescents are on an upward trend (4, 5). Middle school students are experiencing a phase of rapid development accompanied by significant cognitive and emotional changes (6), and these physiological and emotional-cognitive changes heighten susceptibility to conditions such as anxiety and depression, and may precipitate behavioral manifestations of emotional disturbance, including suicide and self-harm (7, 8). In addition, these mental health problems can lead to higher academic difficulties (9), more somatic illness distress (10), and significantly reduce their level of well-being, affecting their life quality. Therefore, fostering the psychological health and *SWB* of middle school students is vital for their positive and healthy development. Accordingly, the present study aims to explore the determinants of *SWB* among middle school students.

## 2 Literature review and research hypotheses

### 2.1 Subjective well-being

*SWB* is a multidimensional construct referring to both cognitive evaluations of life quality and affective experiences (11), and is typically divided into life satisfaction, positive affect, and negative affect (12). Empirical studies have found that *SWB* contributes positively to mental health (13), life quality (14), and life expectancy (15). Previous studies have indicated that *SWB* not only helps to enhance the physical functioning of middle school students (16), but also improves their initiative in academic engagement (17) and strengthens their capacity to manage stressful life events (18). However, research has found that upon entering middle school, students’ life satisfaction decreases (19), positive emotions decrease, and negative emotions increase (20, 21). Therefore, exploring the factors affecting secondary school students’ *SWB* is crucial for promoting their healthy development. Empirical studies suggest that the determinants of *SWB* are broadly classified as either external or internal (22). External influences typically involve social support and family context, while internal influences encompass coping styles and personality traits (23). And among the external factors, family plays a pivotal role in shaping *SWB*.

### 2.2 Parenting styles

Parenting styles represent an amalgamation of emotions, cognitive attitudes, and behavioral patterns parents demonstrate while raising and educating their children (24). Ecosystem theory emphasizes that individuals grow and develop in complex environmental systems, which interact with each other and ultimately influence the individual (25). The family is an important place for the individual to have contact with the outside world at an early stage, and it is the most direct micro-environment that affects the individual, which will have an irreplaceable influence on the individual (26, 27). As important family members, parents significantly impact their children’s emotions, attitudes, perceptions, and behaviors through their behaviors and parenting styles (28–31). Parenting styles have been associated with positive outcomes in one’s mental health (32), with parents adopting positive parenting styles giving their children more warmth, acceptance, and encouragement, while in negative parenting styles, parents are more inclined to show more negativity, punitive discipline, and overprotection (33). Parenting styles are a key determinant of one’s level of *SWB*. Negative parenting styles tend to increase children’s vulnerability to different levels of psychological problems, negative emotional experiences, and unhealthy psychological qualities such as interpersonal sensitivity, anxiety, and depression, which in turn reduce their *SWB*. On the contrary, children who grow up in positive parenting styles exhibit greater overall well-being and psychological functioning (34, 35). However, the pathways linking parenting styles to *SWB* remain largely unclear, and empirical evidence regarding their effects on middle school students remains limited.

### 2.3 Family functioning

Family functioning means the ability of family members to emotionally connect, to communicate, and to work together to deal with external events in the family system, which is characterized by intimacy and adaptability (36). The family environment is a critical determinant of individual growth, as a person’s psychosocial well-being is inextricably linked to the family’s functioning (37). The closer the family members are to each other and the more emotionally connected they are, the better the family functions (38, 39). Good family functioning contributes to the physical health and psychosocial well-being of individuals, mitigates the risk of psychological problems (40), and strengthens their overall life satisfaction and sense of well-being (41). Family dysfunction, such as family conflict, increases the likelihood of anxiety, depression, and other psychological disorders (42). Family functioning, a crucial indicator of the family system, is significantly related to parenting styles. Parental support and encouragement in parenting practices promote affective ties among family members, enhancing family cohesion and intimacy (43). Conversely, control-oriented parenting behaviors negatively correlate with family adaptability and cohesion (44). As important characters within the family system, parents’ parenting approaches and behaviors directly influence their children’s perception of their family atmosphere and environment, ultimately affecting their children’s *SWB* (45). Therefore, Hypothesis 1 (H1) is proposed:

H1: Family functioning mediates the relationship between parenting styles and *SWB*.

### 2.4 Core self-evaluation

Core self-evaluation means an individual’s underlying assessment of their competence and value (46). Judge proposed that core self-evaluation influences individuals via cognitive schema processing of external information (47), and the formation of self-concept affects the processing bias of such information. A well-developed core self-evaluation predisposes individuals to form positive self-perception schemas in their brains, develop positive self-concepts, tend to assess situations positively, trust in their own abilities, and view themselves as competent in controlling developments and solving problems (48). Empirical evidence has established core self-evaluation as a reliable predictor of *SWB* (49, 50). Lower core self-evaluation is associated with higher negative affect, lower positive affect, and reduced life satisfaction (51). Since the evaluation of self-concept is intricately linked to the environment, the core self-assessment is vulnerable to the influence of the external environment (52). The family environment is central to development and helps shape core self-evaluation. Parenting style, in particular, has been identified as a key predictor of core self-evaluation (53). Parental caring and warm parenting behaviors can lead children to develop more positive perceptions and evaluations of themselves, promote children to attribute achievements to themselves, thereby promoting greater self-efficacy and heightened *SWB* (54). Parental harsh and critical parenting behavior tends to undermine children’s self-concept and self-esteem (55). Accordingly, Hypothesis 2 (H2) is advanced.

H2: Core self-evaluation mediates the relationship between parenting styles and *SWB*.

Furthermore, research has documented a robust link between family functioning and core self-evaluation. Positive family contextual resources, such as support from family members, lead individuals to have a more positive view of themselves, thus promoting higher core self-evaluation (56). This study, therefore, posits Hypothesis 3 (H3).

H3: Parenting styles affect *SWB* through the chain mediation of family functioning and core self-evaluation.

**Figure 1:**
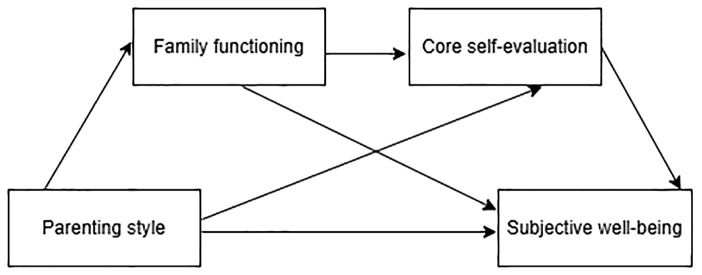
Hypothesized chain mediation model.

Bronfenbrenner’s ecological systems theory posits that human development is shaped not by isolated factors but by multiple nested environmental systems. (25). Within this theoretical framework, the family is the most central and enduring microsystem for the individual. These subsystems are interconnected and interact with one another, collectively maintaining the overall balance of the family ecosystem. Parenting style constitutes a core component of the family system and directly shapes the family atmosphere and parent-child interaction patterns, thereby influencing children’s self-perception and emotional experiences. Therefore, the family, being a key component of the microsystem, exerts considerable influence on how individuals perceive themselves and experience well-being. Based on ecosystem theory and the above research hypotheses, the hypothesized model diagram of this study is as follows (**Figure 1**):

## 3 Methods

### 3.1 Participants

Using cluster sampling, a total of 592 questionnaires were distributed in two middle schools in Anhui Province, China. This study was approved by the school class teacher before the field investigation, and the questionnaire was distributed and collected by the researcher herself, during which the research purpose and precautions were introduced to the students, and informed consent of all participants was obtained. All questionnaires were completed during the classroom study time (10min~20min). After excluding incomplete answers, consecutive repeated answers, and invalid questionnaires with data outside of positive and negative 3 standard deviations, 542 valid questionnaires were retained for analysis. Of these, 282 (52%) were boys, and 260 (48%) were girls. 211 (38.9%) were from urban areas, and 331 (61.1%) were from rural areas. There were 102 (18.8%) only child and 440 (81.2%) non-only child. In terms of grade distribution, the sample included 90 (16.6%) seventh-graders, 74 (13.7%) eighth-graders, and 103 (19.0%) ninth-graders from junior high. The senior high cohort comprised 93 (17.2%) tenth-graders, with 91 (16.8%) in both eleventh and twelfth grade.

### 3.2 Measurement of variables

#### 3.2.1 Parental parenting styles questionnaire

The Chinese version of the Short Egna Minnenav Barndoms Uppfostran (S-EMBU-C) questionnaire, developed by Arrindell (57), and revised by Jiang (58), was adopted. It comprises separate father and mother versions, each containing 21 identical items. These items measure three dimensions: emotional warmth (e.g., “Father/Mother frequently praised me”), rejection (e.g., “Father/Mother’s punishments often exceeded what I deserved”), and overprotection (e.g., “I feel Father/Mother interferes with everything I do”). For this study, parenting dimensions from both parents were combined into one overall measure to examine their predictive role on *SWB*. Positive parenting was indexed by emotional warmth, and negative parenting by rejection and overprotection (59). The scale uses a 4-point Likert scoring scale, with a score of 1 to 4 representing never, occasionally, often, and always, respectively, to calculate the average score in each dimension. The Cronbach’s α coefficients were 0.912 for positive parenting style, 0.865 for negative parenting style, and 0.806 for the total questionnaire.

#### 3.2.2 Family functioning scale

The Chinese version of the Family Adaptability and Cohesion Scales (FACESII-CV), which was developed by Olson (36), and translated and revised by Fei was used (60), consisting of a total of 30 items in two dimensions: intimacy (e.g., “All family members gather together for activities”) and adaptability (e.g., “Each family member participates in making major household decisions”). The scale employs a five-point scoring system, where elevated scores reflect more optimal family functioning. The Cronbach’s α coefficient of the scale in this study was 0.907.

#### 3.2.3 Core self-evaluation scale

The Chinese version of the Core Self-Evaluation Scale (CSES) was developed by Judge (47) and revised by Du (61). The Scale is a unidimensional instrument consisting of 10 items. Each item is rated on a five-point Likert scale, and total scores are computed after reverse-coding relevant items. Higher scores indicate more positive core self-evaluations. The Cronbach’s α coefficient for this scale in this study was 0.866.

#### 3.2.4 Subjective well-being scale

The cognitive component of *SWB* was measured using the Satisfaction with Life scales (SWLS) developed by Diener (62), which has 5 items and uses a seven-point Likert scale, with higher scores representing higher life satisfaction. The Positive Affect and Negative Affect Scale(PANAS), developed by Watson (63) and revised by Qiu (64), was used to measure the emotional component of *SWB*. The scale consists of 18 items on a five-point scale. *SWB* was computed as the sum of standardized life satisfaction and positive affect minus standardized negative affect (65, 66). In this study, the Cronbach’s α coefficients were 0.776 for the Life Satisfaction Scale and 0.825 for the Positive and Negative Affect Scale.

#### 3.3 Statistical analysis

The questionnaire data were entered using Epidata 3.1 and then exported to SPSS 26.0 for data preprocessing (67). All analyses were based on complete data, and missing values were treated with mean substitution. Pearson’s correlation was performed using R 4.5.1 to assess the bivariate associations between variables. Mediation analysis was conducted using Mplus 8.3 with maximum likelihood estimation and 5000 bootstrap resamples to test the chain mediating effects.

## 4 Results

### 4.1 Common method deviation

To assess common method bias, Harman’s single-factor test was conducted. The unrotated factor solution extracted twenty-five factors with eigenvalues exceeding 1, with the first factor accounting for 21.951% of the variance, below the 40% threshold. Thus, no significant common method variance was detected.

### 4.2 Correlation analysis

**Figure 2:**
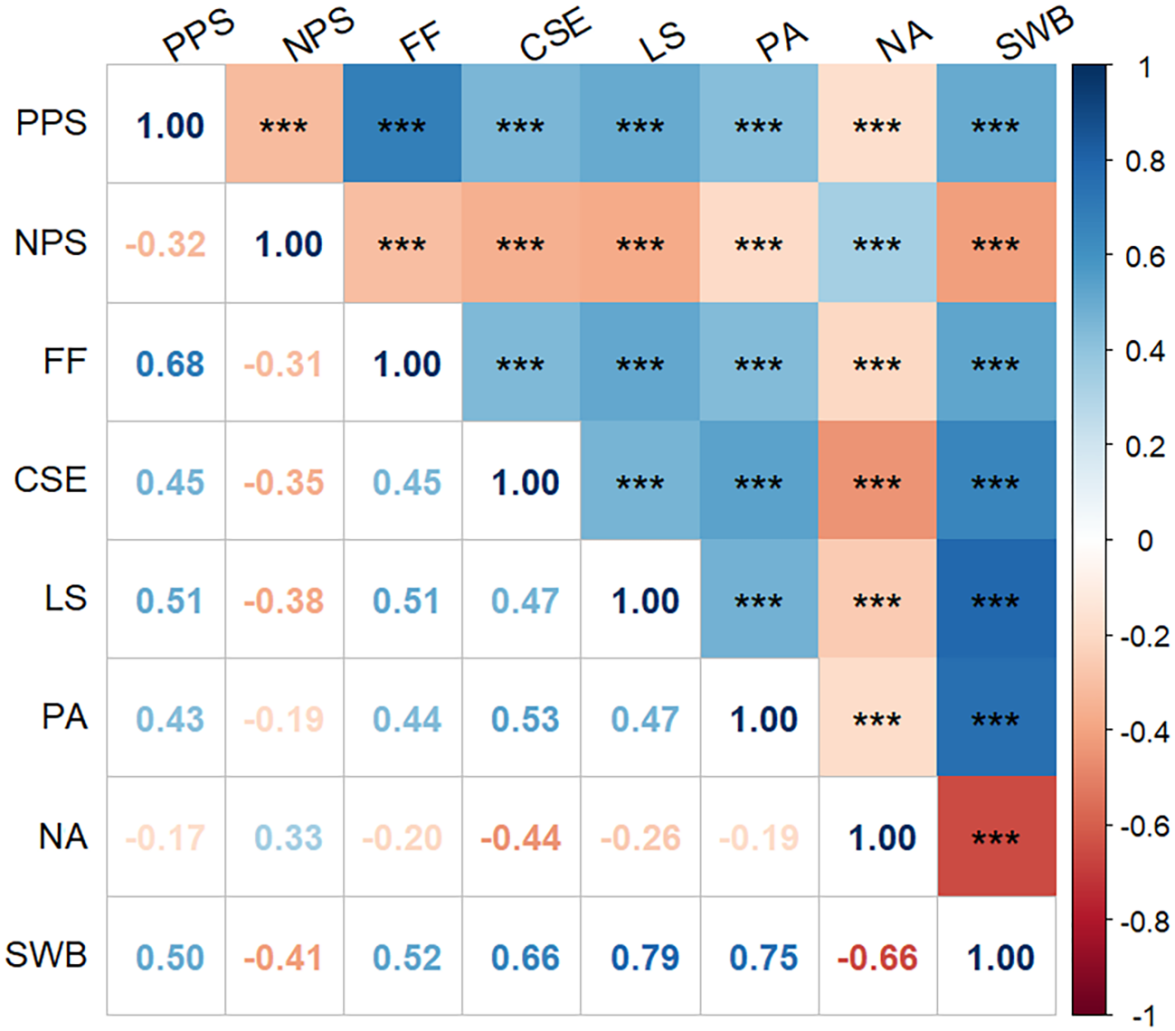
Correlation analysis of parenting styles, family functioning, core self-evaluation, and *SWB*. PPS, positive parenting style; NPS, negative parenting style; FF, family functioning; CSE, core self-evaluation; LS, life satisfaction; PA, positive affect; NA, negative affect; SWB, subjective well-being. *** *p* < 0.01.

Correlation analysis between variables was conducted using Pearson correlation analysis. The correlation coefficients of each variable are shown in **Figure 2**.

As shown in **Figure 2**, positive parenting style was significantly positively correlated with family functioning, core self-evaluation, life satisfaction, positive affect, and *SWB* (*r* = 0.43 ~ 0.68, *p* < 0.001), and significantly negatively correlated with negative affect (*r* = −0.17, *p* < 0.001). Negative parenting style was significantly negatively correlated with family functioning, core self-evaluation, life satisfaction, positive affect, and *SWB* (*r* = −0.41 ~ −0.19, *p* < 0.001), while significantly positively correlated with negative affect (*r* = 0.33, *p* < 0.001). Furthermore, strong positive intercorrelations emerged among family functioning, core self-evaluation, life satisfaction, positive affect, and *SWB* (*r* = 0.44 ~ 0.79, *p* < 0.001), all of which were negatively associated with negative affect (*r* = −0.44 ~ −0.19, *p* < 0.001).

### 4.3 Mediation effect analysis

To further explore the influence of parenting styles on *SWB*, a chain mediation analysis was conducted using Mplus 8.3 with maximum likelihood estimation and 5000 bootstrap resamples to obtain bias-corrected 95% confidence intervals. Positive parenting style and negative parenting style served as independent variables, family functioning and core self-evaluation as mediators, and *SWB* as the dependent variable.

**Figure 3:**
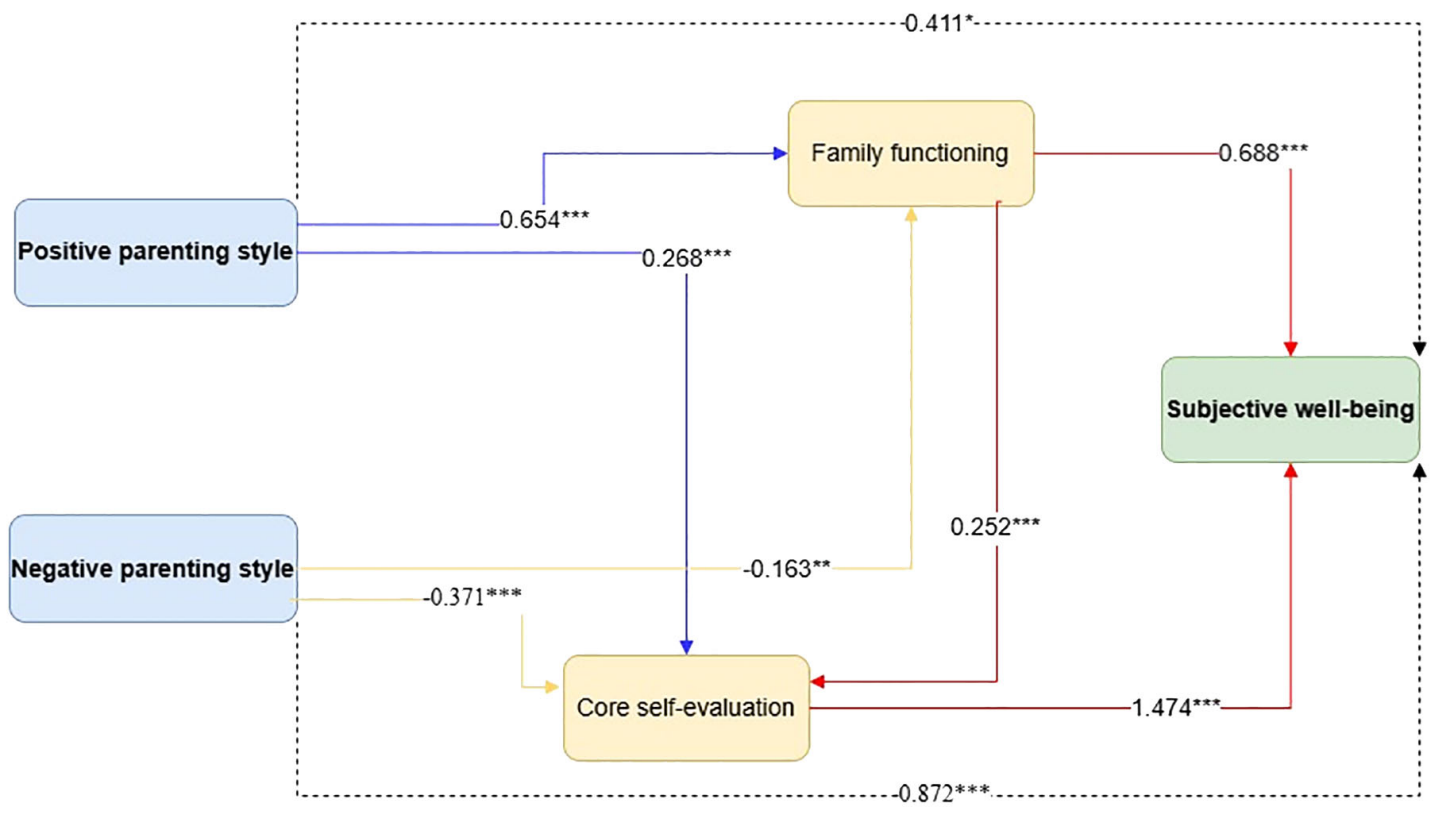
The chain mediating model of family functioning and core self-evaluation on parenting styles and *SWB*. **p < 0.01, ***p < 0.001.

The results of the regression analysis are shown in **Table 1** and **Figure 3**. Positive parenting style positively predicted family functioning (B = 0.654, SE = 0.035, *p* < 0.001) and core self-evaluation (B = 0.268, SE = 0.060, *p* < 0.001). Negative parenting style negatively predicted family functioning (B = −0.163, SE = 0.058, *p* < 0.01) and core self-evaluation (B = −0.371, SE = 0.072, *p* < 0.001). Family functioning significantly positively predicted core self-evaluation (B = 0.252, SE = 0.059, *p* < 0.001). When all variables were entered simultaneously, the direct effect of positive parenting style on *SWB* remained significant (B = 0.411, SE = 0.159, *p* < 0.05), and the direct effect of negative parenting style on *SWB* also remained significant (B = −0.872, SE = 0.200, *p* < 0.001). Family functioning (B = 0.688, SE = 0.162, *p* < 0.001) and core self-evaluation (B = 1.474, SE = 0.116, *p* < 0.001) both significantly positively predicted *SWB*.

**Table 1:** Regression coefficients of the chain mediation model.

Result variable	Predictive variable	B	SE	R^2^
Family functioning	Positive parenting style	0.654^***^	0.035	0.477
	Negative parenting style	−0.163^**^	0.058	
Core self-evaluation	Positive parenting style	0.268^***^	0.060	0.279
	Negative parenting style	−0.371^***^	0.072	
	Family functioning	0.252^***^	0.059	
Subjective well-being	Positive parenting style	0.411^*^	0.159	0.527
	Negative parenting style	−0.872^***^	0.200	
	Family functioning	0.688^***^	0.162	
	Core self-evaluation	1.474^***^	0.116	

* *p* < 0.05, ** *p* <0.01, *** *p* <0.001.

As shown in **Table 2**, the 95% confidence intervals for the total indirect effects of positive and negative parenting styles on *SWB* excluded zero (positive: 1.089, 95% CI [0.831, 1.365]; negative: −0.719, 95% CI [−0.964, −0.482]), indicating that family functioning and core self-evaluation served as mediators in these relationships. Moreover, a significant direct effect of positive parenting style on *SWB* was observed (effect = 0.411, 95% CI [0.092, 0.711]), demonstrating that positive parenting style exerts a direct and positive influence on *SWB*. Both family functioning and core self-evaluation mediated the relationship between positive parenting style and *SWB*, with specific indirect effects of 0.450 and 0.395, respectively. A chain mediation path through both mediators was also significant (effect = 0.243, 95% CI [0.135, 0.372]). For negative parenting style, a significant direct effect on *SWB* was also observed (effect = −0.872, 95% CI [−1.258, −0.478]), indicating that negative parenting style exerts a direct and negative influence on *SWB*. Both family functioning and core self-evaluation mediated the relationship between negative parenting style and *SWB*, with specific indirect effects of −0.112 and −0.546, respectively. A chain mediation path through both mediators was also significant (effect = −0.060, 95% CI [−0.127, −0.020]).

**Table 2:** Bootstrap analysis of the mediation effect test.

Effects	Pathways	Effect value	Boot SE	Bootstrap95%CI	
				LLCI	ULCI
Direct effect	PPS→SWB	0.411	0.159	0.092	0.711
Total indirect effect	PPS→SWB	1.089	0.138	0.831	1.365
Indirect effects	PPS→FF→SWB	0.450	0.109	0.244	0.673
	PPS→CSE→SWB	0.395	0.096	0.220	0.600
	PPS→FF→CSE→SWB	0.243	0.060	0.135	0.372
Direct effect	NPS→SWB	-0.872	0.200	-1.258	-0.478
Total indirect effect	NPS→SWB	-0.719	0.124	-0.964	-0.482
Indirect effects	NPS→FF→SWB	-0.112	0.049	-0.230	-0.034
	NPS→CSE→SWB	-0.546	0.114	-0.775	-0.329
	NPS→FF→CSE→SWB	-0.060	0.027	-0.127	-0.020

PPS, positive parenting style; NPS, negative parenting style; FF, family functioning; CSE, core self-evaluation; LS, life satisfaction; PA, positive affect; NA, negative affect; SWB, subjective well-being.

## 5 Discussion

### 5.1 The direct effect of parenting styles on the *SWB* of middle school students

The results of this study showed that positive parenting styles positively predicted *SWB* and negative parenting styles negatively predicted *SWB*. The family, functioning as a central environment for early personal growth, exerts a profound influence on the formation of a person’s psychological, emotional, and behavioral patterns (68, 69). Positive parenting styles tend to foster favorable developmental outcomes in children and enhance their positive affect, life satisfaction, self-esteem, and academic performance (70, 71). Conversely, children subjected to rejective or overprotective parenting are more likely to exhibit psychological characteristics, including inferiority, heightened sensitivity, and pessimism. Such parenting behaviors tend to undermine the quality of parent–child interactions and intensify relational conflicts, causing individuals to experience more negative emotions, resulting in negative consequences such as physical and mental illnesses and aggressive behaviors (72–74). Moreover, declining fertility intentions have led to a reduction in the number of children per family (75), which may increase the likelihood of parental overindulgence and consequently alter parenting practices, ultimately affecting family well-being. Therefore, in parenting practice, parents should reduce the behaviors of punishing, controlling, and criticizing their children, and give more love, understanding, and support to their children, enhance the harmony of the parent-child relationship, reduce conflicts, enable their children to grow up in a positive and warm family environment, and improve their happiness and *SWB* ultimately.

### 5.2 The mediating role of family functioning between parenting styles and *SWB*

This study demonstrated that parenting styles influence *SWB* through the mediating pathway of family functioning, and H1 was confirmed. Specifically, parenting styles and practices affect the functionality of the family system, thereby influencing the sense of subjective well-being. Family functioning is related to parenting styles, while parenting practices significantly contribute to the formation and maintenance of family functioning (45). When children perceive parental support, family functioning tends to be more effective (76). Morris emphasizes that parenting styles exert a significant influence on shaping the general atmosphere in the family, and negative parenting practices are more likely to create a negative family atmosphere, leading to children becoming more emotional and experiencing more negative emotions. Positive parenting styles create a positive and warm family atmosphere, and children are more emotionally stable (77). Research has found that positive and sincere communication with parents enhances the trust and intimacy between children and parents, and then enhances their experience of family well-being (78, 79). Parental rejection and overprotection can undermine the parent-child bond and impair overall family functioning (80, 81). As fertility intentions decline, the number of children per family has decreased, which may intensify overprotective parenting and disrupt family functioning. Encouraging appropriate fertility levels and optimizing family structure may serve as viable pathways to promote family functioning and children’s psychological well-being, a direction that resonates with China’s current proactive fertility policy. Overall, positive parenting behaviors shape children’s perceptions and experiences of family relationships and atmosphere, which help increase life satisfaction and happiness. Family systems theory posits that the operation of the family unit fundamentally shapes the psychological and emotional wellness of its members. Effective family functioning strengthens an individual’s overall wellness, life satisfaction, and psychological health (82, 83).

### 5.3 The mediating role of core self-evaluation between parenting styles and *SWB*

This study revealed a significant mediating role of core self-evaluation between parenting styles and *SWB*, which validates H2. To be specific, the parenting behaviors affect the level of middle school students’ evaluation of themselves, which consequently affects *SWB*. Core self-evaluation, as a dispositional trait, filters external information via cognitive schemas, which subsequently influences the individual. Cognitive schema is the cognitive structure that influences individuals to recognize themselves and process information about the external environment, and it serves as the primary mechanism through which personality traits shape the processing of external information (84). Parents who employ a nurturing and supportive parenting style tend to provide greater affection and reinforcement, cultivate positive and supportive interactions with their children, so that their children can develop a positive self-perception schema, form a positive self-evaluation, and are more prone to achieve a sense of *SWB* (54). Conversely, individuals experiencing parental control and rejection tend to exhibit negative consequences such as diminished self-esteem and denial of self-value, which results in a weakened core self-evaluation, thereby undermining their *SWB* (85). Furthermore, core self-evaluations can directly influence *SWB* (49). Individuals with high core self-evaluation tend to view their capabilities favorably, recognize their own value, develop positive cognitive schemas, and perceive more well-being in their lives. Individuals with low core self-evaluations, by contrast, often hold negative perceptions of both themselves and the external environment, lack confidence and motivation when facing practical tasks, and fail to experience their sense of value and fulfillment, which leads to lower *SWB* (86).

### 5.4 The chain mediating role of family functioning and core self-evaluation between parenting styles and *SWB*

The results of this study demonstrated a chain mediation effect through family functioning and core self-evaluation between parenting styles and *SWB*, and H3 was validated. Family systems theory conceptualizes the family as an emotional unit defined by the emotional ties and relational dynamics among its members (87). Parenting styles do not influence children in isolation; rather, they shape the emotional atmosphere and systemic functioning of the entire family, thereby further influencing children’s cognitive and emotional states. Meanwhile, positive parenting behaviors, such as good parent-child communication, contribute to the regular functioning of the family and help develop beneficial psychological traits, including optimism and self-confidence (88). A well-functioning family system characterised by close emotional bonds enables children to internalise a positive and valuable self-concept (89). When children grow up in well-functioning families and experience warmth, support, and harmony, they are likely to form positive core self-evaluations, viewing themselves as competent and valuable, which in turn leads to higher levels of *SWB*. Consequently, family functioning and core self-evaluation act as chain mediators between parenting styles and *SWB*.

## 6 Conclusion

This study revealed that parenting styles influence *SWB* both directly and indirectly through the chain mediation of family functioning and core self-evaluation. These findings indicate that interventions should address both family-level and individual-level factors. In practice, parents should adopt warm and supportive parenting approaches, cultivate an atmosphere of open communication and emotional cohesion, and help their children develop positive self-evaluations, thereby enhancing their *SWB*.

## 7 Limitations

This study has several limitations. First, regional variations in economic, educational, cultural, and ethnic contexts may exist. Thus, future research should diversify the sample geographically and incorporate sociocultural factors to test cross-cultural generalizability. Second, longitudinal designs are needed to establish causality.

## Data Availability

The original contributions presented in the study are included in the article/supplementary material. Further inquiries can be directed to the corresponding authors.
